# Progesterone for prevention of preterm birth in women with short cervical length: 2‐year infant outcomes

**DOI:** 10.1002/uog.23126

**Published:** 2021-02-12

**Authors:** C. J. J. Cuijpers, J. Van't Hooft, C. Schneeberger, J. H. Van Der Lee, N. E. Simons, M. A. Van Os, J. Van Der Ven, C. J. M. De Groot, B. W. J. Mol, A. G. Van Wassenaer‐leemhuis

**Affiliations:** ^1^ Department of Obstetrics and Gynecology Amsterdam UMC, Academic Medical Center Amsterdam The Netherlands; ^2^ Department of Infectious Diseases, Amsterdam UMC Academic Medical Center Amsterdam The Netherlands; ^3^ Pediatric Clinical Research Office, Emma Children's Hospital Amsterdam UMC The Netherlands; ^4^ Knowledge Institute of the Dutch Association of Medical Specialists Utrecht The Netherlands; ^5^ Wilhelmina Children's Hospital University Medical Centre Utrecht Utrecht The Netherlands; ^6^ Midwifery Practice Velp Velp The Netherlands; ^7^ Department of Obstetrics and Gynaecology Monash University Clayton Australia

**Keywords:** child, development, preterm birth, progesterone

## Abstract

**Objective:**

To evaluate the long‐term outcomes of children born to women with a short cervix and otherwise low risk for preterm birth, after antenatal exposure to vaginal progesterone *vs* placebo.

**Methods:**

This was a follow‐up study of the Triple P trial, which randomized 80 low‐risk women with a short cervix (≤ 30 mm) at 18–22 weeks' gestation to progesterone (*n* = 41) or placebo (*n* = 39). At 2 years of corrected age, children were invited for a neurodevelopmental assessment, using the Bayley Scales of Infant and Toddler Development, third edition (BSID‐III), and a neurological and physical examination by an assessor blinded to the allocated treatment. Parents filled out the Ages and Stages Questionnaire, the Child Behavior Checklist (CBCL) and a general‐health questionnaire. The main outcome of interest was mean BSID‐III cognitive and motor scores. Additionally, a composite score of mortality and abnormal developmental outcome, including BSID‐III ≤–1 SD, CBCL score in the clinical range and/or parental reported physical problems (at least two operations or at least two hospital admissions in the previous 2 years), was evaluated. Our sample size, dictated by the original sample of the Triple P trial, provided 80% power to detect a mean difference (MD) of 15 points (1 SD) between groups for the BSID‐III tests.

**Results:**

Of the 80 children born to the randomized women, one in the progesterone group and two in the placebo group died in the neonatal period. Follow‐up data were obtained for 59/77 (77%) children and BSID‐III outcomes in 57 children (*n* = 28 in the progesterone group and *n* = 29 in the placebo group) born at a median gestational age of 38 + 6 weeks (interquartile range (IQR), 37 + 3 to 40 + 1 weeks) with a median birth weight of 3240 g (IQR, 2785–3620 g). In the progesterone *vs* placebo groups, mean BSID‐III cognitive development scores were 101.6 *vs* 105.0 (MD, –3.4 (95% CI, –9.3 to 2.6); *P* = 0.29) while mean motor scores were 102.4 *vs* 107.3 (MD, –4.9 (95% CI, –11.2 to 1.4); *P* = 0.13). No differences were seen between the two groups in physical (including genital and neurological examination), behavioral and health‐related outcomes.

**Conclusion:**

In this sample of children born to low‐risk women with a short cervix at screening, no relevant differences in neurodevelopmental, behavioral, health‐related and physical outcomes were found between offspring exposed to vaginal progesterone and those exposed to placebo. © 2020 The Authors. Ultrasound in Obstetrics & Gynecology published by John Wiley & Sons Ltd on behalf of International Society of Ultrasound in Obstetrics and Gynecology.

**CONTRIBUTION**

**What are the novel findings of this work?**

Our study found no evidence of substantial harm, in terms of neurodevelopment and health, in offspring exposed to progesterone in the second and third trimesters of pregnancy.

**What are the clinical implications of this work?**

This work contributes to data that are needed to build reliable evidence on the long‐term safety of *in‐utero* exposure to progesterone, information that is crucial when counseling pregnant women with an indication for progesterone use.

## INTRODUCTION

Preterm birth (PTB) is associated with increased rates of neonatal mortality and long‐term morbidity^1^. The prevention of PTB would therefore substantially benefit infant health and reduce healthcare costs. Women with a short cervix (≤ 30 mm) have a 3‐ to 4‐fold increased risk of PTB^2^. An individual‐patient data (IPD) meta‐analysis evaluating the effect of vaginal progesterone *vs* placebo in 974 women with a singleton pregnancy with a cervical length ≤ 25 mm, showed a reduction in the rate of PTB before 33 weeks' gestation from 22% to 14% (relative risk (RR), 0.62 (95% CI, 0.47–0.81))^3^. This IPD meta‐analysis concluded that there is no evidence that vaginal progesterone has adverse effects on childhood neurodevelopmental outcomes^3^. However, this conclusion is based on the follow‐up data of five randomized trials with a follow‐up period between 2 and 8 years of age and carried out in high‐risk populations (i.e. twin gestation or previous spontaneous PTB), of which four out of the five studies used screening instruments (e.g. questionnaires) instead of in‐person developmental examinations^4^, ^5^, ^6^, ^7^, ^8^. To increase confidence in the absence of harm of progesterone, more sensitive in‐person developmental examination data are needed.

Assessment of long‐term outcomes is particularly important, as previous studies have demonstrated that agents administered to pregnant women with the aim of improving pregnancy outcomes can have unexpected long‐term effects on children, which may not be apparent at birth^9^. Historically, there has been fear of the possibility of masculinization of the genital tract in female fetuses or hypospadias in male infants exposed to progesterone^10^, but several observational studies have been unable to confirm or refute these assumptions^11^, ^12^, ^13^. Studying the developmental outcomes after *in‐utero* exposure to progesterone is important, both in preterm and term infants. First, animal studies suggest that progesterone has a substantial impact on the development of the fetal brain^14^. It is unknown if the same thing happens in humans, but one can hypothesize either a neuroprotective or a deleterious effect by disturbing the brain maturation processes. Second, neurodevelopmental impairment is among the most common complications reported after PTB^15^; thus, it is reasonable to consider that if progesterone is able to decrease the severity of prematurity it could potentially reduce developmental problems.

In the Triple P trial, 80 women with a short (≤ 30 mm) cervix on ultrasonography at 18–22 weeks' gestation, but otherwise at low risk for PTB, were randomized to receive progesterone or placebo^16^. The aim of this follow‐up study was to compare the neurodevelopmental and other health outcomes of their infants at 2 years of corrected age using robust diagnostic instruments.

## METHODS

The Triple P trial was a multicenter double‐blind placebo‐controlled randomized trial on the effectiveness of vaginal progesterone in reducing adverse neonatal outcome through a reduction in the rate of PTB in women with a low‐risk pregnancy and a short cervical length (Registration No: NL1961). The trial and its follow‐up were approved by the Medical Ethics Committee of the Amsterdam UMC, Academic Medical Centre, The Netherlands (AMC 08‐328), and its protocol, including the plans for follow‐up, was published in advance^17^.

The trial was discontinued early owing to the unexpectedly low number of women with a short (≤ 30 mm) sonographic cervix at two independent assessments performed within a fortnight. Between 2009 and 2013 a total of 20 234 women were screened, of whom 151 were eligible for inclusion and 80 of these agreed to participate. After providing informed consent, women were randomized to progesterone (*n* = 41) or placebo (*n* = 39). The study medication was self‐administered vaginally on a daily basis between 22 and 34 weeks' gestation, using capsules of 200 mg micronized progesterone or identical‐appearing placebo capsules. The primary outcome measure in the original trial was a composite of adverse neonatal outcomes until 10 weeks after the expected date of delivery. Further details are provided in van Os *et al*.^16^. Adverse neonatal outcome occurred in 5% of women in the progesterone group *vs* 11% in the control group (RR, 0.47 (95% CI, 0.09–2.4)). PTB before 32 weeks occurred in 2% in the progesterone group *vs* 8% in the control group (RR, 0.33 (95% CI, 0.04–3.0)). Although adherence to the study medication was moderate (only 51% used ≥ 80% of the study medication), exposure to the medication could be up to 12 weeks, as the majority of the women in the progesterone group (88%) delivered after 34 weeks' gestation.

### Follow‐up assessment

All families with a living child who participated in the original Triple P trial were contacted by phone 3 months prior to the corrected age of 2 years, corrected age being defined as the age calculated from the due date. After the parents had provided informed consent, the cognitive and motor development of the infants was assessed using the cognitive and motor scales of the Bayley Scales of Infant and Toddler Development, third edition (BSID‐III), followed by a physical and neurological examination. A trained team of psychologists and medical doctors performed all Bayley tests at home or in an outpatient clinic. Parents were asked to fill out the questionnaires before or shortly after the visit. Parents, psychologists, pediatricians and researchers involved in data collection and entry remained blind to the allocated treatment. The main outcome of interest in this follow‐up study was mean BSID‐III cognitive and motor scores.

The Dutch version of the BSID‐III was used to assess cognitive and motor development^18^, but we used the BSID‐III norms for children of the US population (mean of 100 and SD of 15)^19^, as the Dutch norms were not yet available. The test and its norms are used worldwide in healthcare settings, as well as for scientific research purposes. A score of ≤ 70 represents severe neurodevelopmental impairment. A score of 70 to ≤ 85 points (i.e. > 1 SD below the mean) for any of the scales of the BSID‐III represents mild impairment and is often used to identify children in need of intervention.

The parents were asked to complete a general‐health questionnaire comprising sociodemographic characteristics of the parents and the child and clinical history of the child after initial discharge from hospital and up to the age of 2 years, including use of medication, hospital admissions and need for surgery. Physical and neurological examination was performed by one of three medical doctors (A.G.v.W.‐L., C.S. and C.J.J.C.) using a standardized assessment format evaluating vision, hearing, heart, lung, abdomen, dermal, genital and neurological abnormalities. Physical abnormalities were assessed and combined into categories such as congenital abnormalities, syndromes/genetic disorders and neurological abnormalities. If more than one abnormality in one category was found in the same child, this was counted as one case. Particular attention was given to all abnormalities in the genital region.

Two validated parental questionnaires were used: the Ages and Stages Questionnaire (ASQ) third edition and the Child Behavior Checklist for ages 1.5–5 years (CBCL)^20^, ^21^. The ASQ is a developmental screening tool that covers five domains of child development: communication, gross motor and fine motor development, problem‐solving and personal–social skills. A validated Dutch translation of the ASQ 24 months was used. ASQ scores of 1 SD below the normative mean in two or more domains, or 2 SD below the normative mean in at least one domain were scored as abnormal, consistent with the clinical use of the ASQ in The Netherlands^22^. The ASQ was added to the protocol in order to gain additional information on child development from a parental perspective. The CBCL assesses the parental perception of social competency and behavioral problems during the previous 2 months. It informs on eight subscales: emotionally reactive, anxious or depressed, somatic complaints, withdrawn, sleep problems, attention problems and aggressive behavior. Data from these subscales can be summed to provide a combined total‐problems score and two broad‐band scale scores (internalizing problems and externalizing problems). A score > 97^th^ percentile on any of the subscales or a score > 90^th^ percentile in one of the two broad‐band scales or total‐problems score was defined as abnormal and clinically relevant, indicating serious behavioral problems (clinical range), a cut‐off that is also consistent with clinical use in The Netherlands^21^.

Abnormal scores in any of the three developmental assessment tools (BSID‐III cognitive or motor score ≤–1 SD; CBCL score in the clinical range; or physical problems defined as the need for two or more operations or two or more hospital admissions in the 2 years prior to the assessment) were combined as a binary outcome of ‘abnormal developmental outcome’. The abnormal developmental outcome was combined with mortality to demonstrate the entire spectrum of adverse outcomes from randomization until follow‐up at 2 years of age. Research nurses in the participating centers cross‐checked the medical records of all children who participated in the original trial to track the possible occurrence of death from birth until the age of 2 years. This allowed us to obtain largely reliable mortality data from non‐responders as well.

### Power calculation

The main outcomes of interest were the BSID‐III cognitive and motor scores. An indicative power calculation showed that 17 children per group would give 80% power to find a mean difference of 15 points in the BSID‐III scores (mean of 100 and SD of 1) with a two‐sided significance level of 0.05. Detection of a more subtle (but clinically relevant) difference of 7.5 points in the mean (corresponding to 0.5 SD) with 80% power would require 64 children per group. Because the size of the study was predefined by the number of women recruited to the Triple P trial, the study was deemed sufficiently powered to demonstrate a difference of 1 SD in the means in the BSID‐III test between the two groups, but was underpowered to detect smaller (but clinically relevant) differences.

### Statistical analysis

Differences in baseline characteristics of the mothers and children participating in the Triple P follow‐up study between those who were exposed to vaginal progesterone and those who were not, as well as differences between participants in the original trial who participated in this follow‐up study and those who were lost to follow‐up, were compared using the unpaired *t*‐test, Mann–Whitney *U*‐test, χ‐square test or Fisher's exact test, as appropriate.

Mean cognitive and motor scores of the BSID‐III, as well as the proportion of children with a mild (corresponding to –1 SD or 15 points below the mean) or severe (corresponding to –2 SD or > 30 points below the mean) cognitive and motor impairment were calculated. All other test outcomes (neurological examination, general physical examination, genital examination, ASQ and CBCL questionnaires) were reported as binary outcomes. Results of the general‐health questionnaire (providing information on the need for medical specialist and/or developmental care, use of medication in the past and present, hospital admission and need for surgery) were clustered into binary categories to distinguish between frequent use (e.g. more than one hospital admission and/or more than one operation) and ‘normal’ use of healthcare services. These cut‐offs were all predefined before performing the analysis, as they have been used by our research team in previous follow‐up studies using the same questionnaire^23^.

Potential confounders were visualized using a directed acyclic graph. The graph demonstrated no potential confounders and therefore no correction was applied (Figure S1). The unpaired *t*‐test, Mann–Whitney *U*‐test, χ‐square test or Fisher's exact test was used for comparison of outcomes between the progesterone and placebo groups, with a significance level of 0.05 for a two‐tailed test. Analysis was performed according to the intention‐to‐treat principle.

For the composite outcome, combining mortality and abnormal developmental outcome, we used imputed data for the children that were lost to follow‐up. This was necessary to keep the denominator consistent with the original sample of the Triple P trial (*n* = 80). We imputed all missing outcomes in children that were lost to follow‐up using multiple imputation techniques with 10 datasets using the following variables as predictors: ethnicity, maternal age, smoking at start of pregnancy, parental education and neonatal outcomes, comprising gestational age at birth, birth weight, neonatal sepsis, infant respiratory distress syndrome, intraventricular hemorrhage, necrotizing enterocolitis, gender of the neonate. Odds ratios (ORs) were calculated using generalized linear models. SPSS version 25 (IBM Inc., Armonk, NY, USA) was used for all analyses^24^.

## RESULTS

Of the 80 women and children enrolled in the Triple P trial (41 in the progesterone group and 39 in the placebo group), three children (one in the progesterone group and two in the placebo group) died in the neonatal period owing to extreme prematurity (median gestational age, 24 + 0 weeks), leaving 77 surviving children eligible for follow‐up. All 77 children were alive at the age of 2 years. Follow‐up data were collected between August 2012 and December 2015.

Eighteen (23%) children were lost to follow‐up, either because there was no contact information available (seven in the progesterone group and four in the placebo group) or they declined further participation (four in the progesterone group and three in the placebo group). The pregnancy and neonatal outcomes and background characteristics of the 59 participants of the follow‐up study were broadly similar to those of the 18 who were lost to follow‐up, except for a higher loss to follow‐up of parents with low‐level education (6/18 (33%) *v*s 11/59 (19%); *P* < 0.001) and of non‐white‐European ethnic origin (10/18 (56%) *vs* 14/59 (24%); *P* = 0.01) (Table S1).

**Figure 1:**
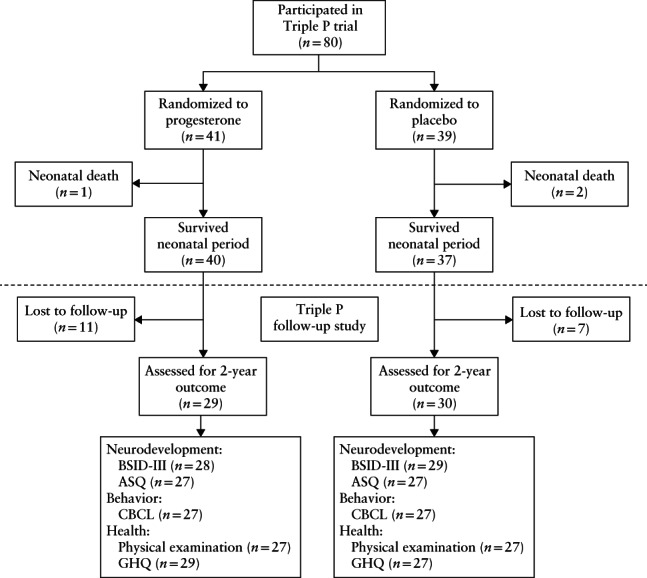
Flowchart showing participants of original Triple P trial who took part in current follow‐up study at corrected age of 2 years. ASQ, Ages and Stages Questionnaire, third edition; BSID‐III, Bayley Scales of Infant and Toddler Development, third edition; CBCL, child behavior checklist; GHQ, general‐health questionnaire.

Of the 59/77 (77%) children who participated in the follow‐up study, 57/77 (74%) underwent BSID‐III assessment (28 in the progesterone group and 29 in the placebo group) and 54/77 (70%) underwent a physical examination (Figure 1). Participants were assessed at a median corrected age of 25 months (interquartile range (IQR), 23–27 months). Median gestational age at birth was 38 + 6 weeks (IQR, 37 + 1 to 40 + 2 weeks) in the progesterone group and 38 + 5 weeks (IQR, 37 + 6 to 40 + 1 weeks) in the placebo group (Table 1). Other relevant maternal and infant characteristics and pregnancy and neonatal outcomes were comparable between the progesterone and placebo groups (Table 1).

**Table 1:** Pregnancy and neonatal outcomes and sociodemographic characteristics of 57 mothers and their children who participated in Triple P follow‐up study and underwent BSID‐III assessment at 2 years of age, according to whether mothers were randomized to progesterone or placebo in Triple P trial

Parameter	Progesterone (*n* = 28)	Placebo (*n* = 29)	*P*
Maternal characteristics			
Age at randomization (years)	31 (26–34)	31 (29–34)	0.46
Nulliparous	22 (79)	18 (62)	0.17
Parental education*			0.94
High	17 (61)	18/28 (64)	
Middle	5 (18)	5/28 (18)	
Low	6 (21)	5/28 (18)	
White European	22 (79)	22 (76)	0.81
Infant characteristics at 2 years			
Living in two‐parent family†	25 (89)	26/27 (96)	0.51
First‐born child	22 (79)	16/27 (59)	0.12
Dutch main language spoken at home	26 (93)	21/27 (78)	0.11
Bilingual	7 (25)	9/27 (33)	0.50
Breastfed for first 6 months‡	6/27 (22)	9/27 (33)	0.36
Use of day care	19 (68)	18/27 (67)	0.32
Pregnancy outcome			
Corticosteroids during pregnancy	5 (18)	5 (17)	0.95
PPROM	4 (14)	3 (10)	0.71
Complied with treatment§	20 (71)	15/28 (54)	0.21
Neonatal outcome			
Male gender	19 (68)	13 (45)	0.08
Composite adverse neonatal outcome¶	0 (0)	2 (7)	0.49
Admission to NICU	0 (0)	5 (17)	0.05
GA at birth (weeks)	38 + 6 (37 + 1 to 40 + 2)	38 + 5 (37 + 6 to 40 + 1)	0.82
GA at birth < 32 weeks	0 (0)	3 (10)	0.24
GA at birth < 34 weeks	2 (7)	3 (10)	1.00
GA at birth < 37 weeks**	5 (18)	4 (14)	0.73
Birth weight (g)	3013 (2603–3506)	3360 (2915–3755)	0.13
Birth weight < 2500 g	5 (18)	4 (14)	0.73
Birth weight < 1500 g	0 (0)	2 (7)	0.49

Data are given as median (interquartile range), *n* (%) or *n*/*N* (%).

* Parental education: ‘low level’ (< 6 total years postelementary schooling) if at least one parent had a low level of education (but not if one parent had a high level); ‘middle level’ (6–8 total years postelementary schooling) if both parents had middle level of education; ‘high level’ (> 8 total years of postelementary schooling) if at least one parent was highly educated.

† Two biological parents or one biological and one non‐biological parent.

‡ Breastfeeding exclusively or in combination with formula for at least 6 months.

§ Used ≥ 80% of study medication.

¶ Composite adverse neonatal outcome until 10 weeks after expected date of delivery, containing following components: respiratory distress syndrome, bronchopulmonary dysplasia, intracerebral hemorrhage > Grade II, necrotizing enterocolitis > Stage 1, proven sepsis and death before discharge.

** Total number of children born < 37 weeks, i.e. includes children born < 34 weeks and < 32 weeks.

BSID‐III, Bayley Scales of Infant and Toddler Development, third edition; GA, gestational age; NICU, neonatal intensive care unit; PPROM, preterm prelabor rupture of membranes.

### Neurodevelopmental outcomes

The mean BSID‐III cognitive score did not differ significantly between the two groups, being 101.6 in the progesterone *vs* 105.0 in the placebo group (mean difference, –3.4 (95% CI, –9.3 to 2.6); *P* = 0.29). Similarly, the BSID‐III motor scores did not differ significantly between the two groups (Table 2). The proportion of children with a BSID‐III score (either cognitive or motor) of ≤–1 SD was comparable between the two groups (1/28 *vs* 1/29) (Table 3). Neurological examination showed no cases of cerebral palsy, and the occurrence of mild neurological abnormalities, consisting of mild hypotonia or hypertonia of the extremities, was low and not statistically significantly different between the progesterone and placebo groups (8% *vs* 4%; RR, 2.00 (95% CI, 0.19–20.67)). An abnormal CBCL score was found in one child (1/27 (4%)) in the progesterone group and in four (4/27 (15%)) in the placebo group (RR, 0.22 (95% CI, 0.02–2.12)). The number of children with abnormal ASQ scores was comparable between the two groups (Table 2).

**Table 2:** Neurodevelopmental, physical, behavioral and health‐related infant outcomes at 2 years corrected age, according to whether they were exposed to progesterone *in utero*

Outcome	Progesterone (*n* = 30)	Placebo (*n* = 32)	RR or mean difference (95% CI)	*P*
BSID‐III*				
Cognitive composite score	101.6 ± 9.7	105.0 ± 12.5	–3.4 (–9.3 to 2.6)	0.29
Motor composite score	102.4 ± 10.9	107.3 ± 12.6	–4.9 (–11.2 to 1.4)	0.13
Fine motor mean scale scores	11.4 ± 2.3	12.8 ± 2.7	–1.4 (–2.7 to –0.9)	0.04
Gross motor score	9.2 ± 2.0	9.5 ± 2.2	–0.3 (–1.4 to 0.8)	0.58
Neurological examination				
Mild abnormality	2/25 (8)	1/25 (4)	2.00 (0.19 to 20.67)	1.00
General physical examination†				
Congenital abnormality	7/26 (27)	3/26 (12)	2.33 (0.68 to 8.05)	0.29
Minor‡	6/26 (23)	3/26 (12)	2.00 (0.56 to 7.16)	0.47
Major§	1/26 (4)	0/26 (0)	—	1.00
Syndrome/genetic disorder†	0/27 (0)	1/27 (4)	—	1.00
Genital abnormality¶	3/26 (12)	3/25 (12)	0.96 (0.21 to 4.3)	1.00
Questionnaires				
Abnormal ASQ**	5/27 (19)	5/27 (19)	1.00 (0.33 to 3.06)	1.00
Abnormal CBCL††	1/27 (4)	4/27 (15)	0.22 (0.02 to 2.12)	0.35
General health				
Need for healthcare providers additional to visits to GP (developmental support and/or specialist care)	14/29 (48)	15/27 (56)	0.87 (0.52 to 1.44)	0.59
Use of any medication in 2 years after birth	21/29 (72)	22/27 (81)	0.89 (0.66 to 1.19)	0.42
At least two hospital admissions in 2 years after birth	2/29 (7)	2/25 (8)	0.86 (0.13 to 5.68)	1.00
At least two operations in 2 years after birth	0/29 (0)	1/27 (4)	—	1.00

Data are given as mean ± SD or *n*/*N* (%).

* Data available for: 29 infants in placebo group; 28 infants for cognitive composite score and fine motor score and 27 infants for motor composite score and gross motor score in progesterone group.

† Data available for: 27 infants in placebo group; 27 infants in progesterone group.

‡ Progesterone group: hemangioma (*n* = 1), combination of café‐au‐lait spot and small cardiac septal defect (*n* = 1), combination of two dimples on back and café‐au‐lait spots (*n* = 1), depigmented small stripes on upper body (*n* = 1), color differences between two irises (*n* = 1), small umbilical hernia (*n* = 1); placebo group: hemangioma (*n* = 1), isolated café‐au‐lait spots (*n* = 1), ptosis in one eye (*n* = 1).

§ Combination of failure to thrive, need for percutaneous endoscopic gastrostomy tube and genital abnormality (small testes and thin penis with normal length) with no known underlying genetic cause at time of writing.

¶ Progesterone group: small testes and thin penis with normal length (*n* = 1), café‐au‐lait spot of 5 cm on labia majora (*n* = 1), underdeveloped scrotal skin (*n* = 1); placebo group: undescended testis (*n* = 1), unretractable foreskin (*n* = 1), labial adhesion to 70% of labium minus (*n* = 1).

** Defined as score 1 SD below normative mean on two or more domains, or as score of 2 SD below normative mean on at least one domain.

†† Defined as score in the clinical range (> 97^th^ percentile).

ASQ, Ages and Stages Questionnaire, third edition; BSID‐III, Bayley Scales of Infant and Toddler Development, third edition; CBCL, Child Behavior Checklist; GP, general practitioner; RR, relative risk.

**Table 3:** Complete‐case and multiple‐imputation analyses for composite outcome of mortality and abnormal development in 80 infants included in Triple P trial, from time of randomization until 2 years of corrected age

Outcome	Progesterone	Placebo	Odds ratio (95% CI)	*P*
Complete‐case analysis				
*n*	41	39		
Neonatal death	1/41 (2)	2/39 (5)	0.46 (0.04–5.32)	0.53
Death up to 2 years of age	1/41 (2)	2/39 (5)	0.46 (0.04–5.32)	0.53
No follow‐up	11/40 (28)	7/37 (19)	0.62 (0.21–1.80)	0.37
Assessment at 2 years of age				
*n*	29	30		
BSID‐III (cognitive or motor)				
≤–1 SD*	1/28 (4)	1/29(3)	1.04 (0.06–17.43)	1.00
≤–2 SD	0/28 (0)	0/29 (0)	—	—
Abnormal CBCL†	1/27 (4)	4/27 (15)	0.22 (0.02–2.12)	0.35
Physical problem‡	3/29 (10)	2/27 (7)	1.44 (0.22–9.37)	1.00
Abnormal developmental outcome§	5/29 (17)	5/30 (17)	0.97 (0.31–2.99)	1.00
Death or abnormal developmental outcome	6/41 (15)	7/39 (18)	0.78 (0.24–2.58)	0.69
Multiple imputation¶				
*n*	41	39		
BSID‐III (cognitive or motor) ≤–1 SD*	6.4/40 (16)	4.7/37 (13)	0.77 (0.17–3.60)	0.74
Abnormal CBCL†	6.4/40 (16)	7.7/37 (21)	1.37 (0.41–4.55)	0.61
Physical problem‡	3.8/40 (10)	4.3/37 (12)	1.17 (0.24–5.70)	0.85
Abnormal developmental outcome§	13.4/40 (34)	11.7/37 (32)	0.94 (0.45–1.99)	0.87
Death or abnormal developmental outcome**	14.4/41 (35)	13.7/39 (35)	1.00 (0.50–1.98)	0.99

Data are given as *n*/*N* (%) unless otherwise indicated.

* Normal or abnormal scores were based on mean score (100) and SD of predefined reference group.

Abnormal BSID‐III is defined as score ≤ –1 SD below normative mean (100).

† Defined as score in clinical range (> 97^th^ percentile).

‡ Defined as at least two hospital admissions and/or at least two operations in 2 years after birth.

§ Composite of BSID‐III score ≤–1 SD or CBCL score in clinical range or physical problem (at least two hospital admissions and/or at least two operations in 2 years after birth).

¶ Pooled outcomes of 10 imputed datasets.

** Composite score of neonatal death (no imputed data) and abnormal developmental outcome (with imputed data). BSID‐III, Bayley Scales of Infant and Toddler Development, third edition; CBCL, Child Behavior Checklist.

### Physical outcomes

Miscellaneous minor congenital malformations were twice as frequent in the progesterone group than in the placebo group, but this difference was not statistically significant (6/26 (23%) *vs* 3/26 (12%); RR, 2.0 (95% CI, 0.56–7.16)). This included malformations such as hemangioma, cafe‐au‐lait spot, color difference between the two irises, ptosis in one eye, umbilical hernia and small cardiac septal defect (Table 2). No consistent differences in the type of congenital malformation could be detected between the groups. No differences in genital malformations or other health‐related outcomes were seen between the progesterone and placebo groups (Table 2). There was one child with a genetic disorder, Noonan syndrome, in the placebo group.

### Composite abnormal developmental outcome and mortality

Composite abnormal developmental outcome (consisting of abnormal BSID‐III or CBCL score or physical impairment) was found in 5/29 (17%) children in the progesterone group and 5/30 (17%) children in the placebo group (OR, 0.97 (95% CI, 0.31–2.99)) (Table 3). After multiple imputation, the composite abnormal developmental outcome doubled in both groups, to 34% in the progesterone group and 32% in the placebo group (OR, 0.94 (95% CI, 0.45–1.99)), but remained similar between the groups. Comparable results were found when combining abnormal developmental outcome with mortality in a multiple imputed dataset (Table 3).

## DISCUSSION

In this 2‐year follow‐up study of a randomized clinical trial comparing antenatal progesterone with placebo for the prevention of preterm birth in low‐risk women with a mid‐trimester short cervix, we found no significant differences in neurodevelopmental outcomes assessed by the Bayley‐III scale. No differences were found in the other physical, behavioral and health‐related outcomes in children at a corrected age of 2 years.

This study has several strengths. First, as it was a follow‐up study of a randomized controlled trial, it was possible to maintain blinding of the parents, care‐providers and researchers to the treatment allocation during the follow‐up measurements and data entry. This prevented performance and detection bias. Second, instead of parental reports only, this follow‐up study used a broad variety of validated instruments and assessments such as the BSID‐III test and neurological and physical assessment by medical doctors. To our knowledge, only one other randomized trial (the OPPTIMUM study^5^) has reported BSID outcomes and also physical assessment results in children exposed to progesterone *vs* placebo during pregnancy. A third strength of our study is the relatively long *in‐utero* exposure to progesterone compared with that of other studies. In the Triple P trial, women started using daily progesterone at 22 weeks' gestation and were able to continue until 34 weeks, as the vast majority of women delivered at term. Irrespective of differences in compliance, children in our study had a potentially longer *in‐utero* exposure to progesterone than did those in other studies^3^. This is important in case an association between duration of *in‐utero* exposure to progesterone and potential harms or benefits is found in the future.

The main limitation of this study was the small number of patients randomized in the original Triple P study and therefore low statistical power to explore more subtle but clinically relevant effects. Although this follow‐up study had enough power to detect a 1 SD (15 points) mean difference in BSID‐III scores between the two groups, it is unlikely that such a large difference due to exposure to progesterone would have been found. The study was not powered to detect a smaller but clinically important difference of 0.5 SD (7.5 points). The same holds true for the other outcome measures reported. Another limitation of the study is the loss to follow‐up. Even though a total follow‐up rate of 77% (and 74% for BSID‐III participants) is quite high for a long‐term follow‐up study, there is a risk of attrition bias. In this study, this is probably the case, as there was a difference in parental education and ethnicity between the participants of this follow‐up study and those who were lost to follow‐up. The same applies to neonatal adverse outcome, prematurity and low birth weight though the difference was not significant (Table S1). As these factors are strongly associated with neurodevelopment^25^, ^26^, ^27^, ^28^, the relatively ‘low‐risk’ sample at follow‐up could have reduced differences between the two groups. We tried to overcome this attrition bias by performing multiple imputation, in which factors such as education and ethnicity were used as predictors for the imputed neurodevelopmental variables. It is therefore not surprising that the multiple imputation analysis showed a two‐fold increase in the rate of neurodevelopmental problems in both groups, reaching values similar to the rates seen in the OPPTIMUM follow‐up study^5^, nevertheless there was no statistically significant difference in this parameter between the placebo and progesterone groups. Attrition bias is a limitation of most follow‐up studies and hampers the generalizability of results.

Prophylactic administration of vaginal progesterone is a common practice globally for women with a history of spontaneous PTB^29^. The use of progesterone for the prevention of PTB in other populations (i.e. low‐risk women with a short cervix) is still debated, and variation in practice is high. The 2013 Cochrane review on progesterone for the prevention of PTB concluded that there was a positive effect on neonatal outcome, but recommended that obtaining long‐term follow‐up data should be a priority^29^. This conclusion has become even more relevant with new insights into the effects of progesterone on the developing brain in animal models^14^. Owing to its lipophilic structure, progesterone can cross the blood–brain barrier, and it has been shown that it plays a role in neuronal proliferation and differentiation, myelination and brain sexual differentiation^14^. Our Triple P follow‐up study contributes to the knowledge of long‐term infant outcomes after *in‐utero* exposure to progesterone.

Findings from the Triple P follow‐up study are consistent with previous follow‐up studies showing no significant signs of harm with respect to neurodevelopment, hospitalization or mortality in children exposed to progesterone compared with placebo^4^, ^5^, ^6^, ^7^, ^8^. However, our findings do not confirm a significant increase in renal, gastrointestinal or respiratory impairment found in the secondary analysis of the OPPTIMUM trial (e.g. renal‐impairment RR, 3.65 (95% CI, 1.96–6.82)), although with low absolute rates of 1 to 2%^5^. We were also unable to confirm the 8‐fold increased risk of cardiac abnormalities reported in the follow‐up study of the PREDICT trial at 8 years of age^8^. The non‐significant 4‐fold decrease in abnormal CBCL scores in the progesterone group observed in our study has not been reported in other follow‐up studies, and was probably caused by a type‐I error related to our small sample size and multiple testing. However, there is also a possibility that it could be related to a potential neuroprotective mechanism of progesterone *in utero*. If this were true, the observed difference in behavioral problems between the progesterone and placebo groups could be clinically relevant and therefore valuable for hypothesis‐generating in future follow‐up studies on the use of progesterone.

It remains possible that the potential impact of progesterone on the development of the brain has not been revealed by the measurements used in previous follow‐up studies (including OPPTIMUM and our follow‐up study), as children might have been too young to study the possible side‐effects of antenatal exposure to progesterone. Developmental disabilities become more apparent at around the age of 5 years, and are a better predictor of performance in later life^30^, ^31^. Therefore, we strongly encourage researchers who carried out randomized trials on progesterone to perform follow‐up studies at later ages using appropriate diagnostic outcome measures.

In conclusion, in mostly (near) term‐born offspring of women with a mid‐pregnancy short cervix, physical, behavioral and neurodevelopmental outcomes at a corrected age of 2 years were not found to be different between children exposed *in utero* to progesterone *vs* those exposed to placebo. Antenatal exposure to progesterone does not seem to indicate major concerns for the neurodevelopment and health of infants at 2 years of age. Our data should contribute to future meta‐analyses to increase confidence that progesterone can be considered safe to use in the second and third trimesters of pregnancy.

## Supporting information


**Figure S1** Directed acyclic graphs of causal assumption in Triple P follow‐up study.
**Table S1** Pregnancy and neonatal outcomes and baseline characteristics of mothers and their children who participated in Triple P follow‐up study and those who were lost to follow‐up

## Data Availability

The data that support the findings of this study are available on request from the corresponding author. The data are not publicly available due to privacy or ethical restrictions.
