# Supplementary material for: Progesterone for prevention of preterm birth in women with short cervical length: 2‐year infant outcomes
Source: Ultrasound Obstet Gynecol. 2021 Feb 12;57(3):431–9. doi: 10.1002/uog.23126 (PMC7986902; doi:10.1002/uog.23126)
Supplement: Supplementary file 1 — Figure S1 Directed acyclic graphs of causal assumption in Triple P follow‐up study. Table S1 Pregnancy and neonatal outcomes and baseline characteristics of mothers and their children who participated in Triple P follow‐up study and those who were lost to follow‐up [file UOG-57-431-s001.docx]

**Figure S1.** Directed Acyclic Graphs of causal assumption in the Triple P follow-up study. The direction of the arrows shows which variable will affect another variable, thus showing a potential causal pathways and possible confounders. In this study no confounders could be identified (e.g. there are no arrows that point out to ‘randomisation’ or ‘vaginal progesterone’).

**
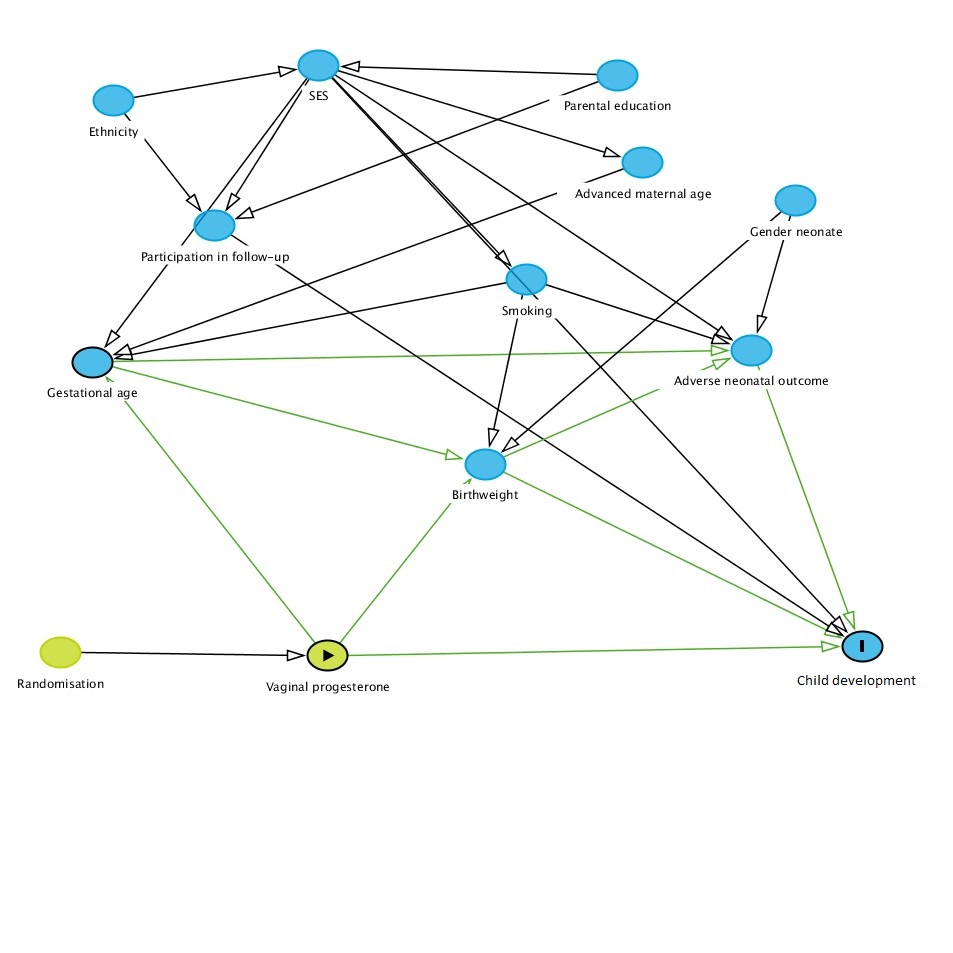
**

**Table S1.** Pregnancy and neonatal outcomes and baseline characteristics of mothers and their children who participated in Triple P follow-up study and those who were lost to follow-up

|  | **Mothers and their children that were assessed at two years follow-up or lost to follow-up** | | |
| --- | --- | --- | --- |
| **Baseline characteristics mothers** | **Follow-up=59** | **Lost to follow-up N=18*** | **P value** |
| Median (IQR) maternal age at randomization | 31 (24-38) | 29 (19-39) | 0.65 |
| Median (IQR)Gestational age | 38+5 (36+0 - 41+3) | 39+0 (36+1 -41+6) | 0.56 |
| Nulliparity n(%) | 41 (69.5) | 11 (61.1) | 0.51 |
| Parental education ‡  High n(%)  Middle n(%)  Low n(%) | 37 (63.8)  10 (17.2)  11 (19.0) | 0 (0.0)  1 (5.6)  6 (33.3) | 0.00 |
| Ethnic origin white European n(%) | 45 (76.3) | 8 (44.4) | 0.01 |
| Smoking n(%) | 11 (21.2) | 5 (29.4) | 0.51 |
| **Pregnancy outcomes** |  |  |  |
| Corticosteroids during pregnancy n(%) | 10 (16.9) | 5 (25.0) | 0.73 |
| PPROM n(%) | 7 (11.9) | 2 (11.1) | 1.00 |
| Treatment compliance (n taken >80% of medication) n(%) | 37 (63.8) | 11 (73.3) | 0.56 |
| **Neonatal outcomes** |  |  |  |
| Gender male n(%) | 33 (55.9) | 10 (55.6) | 1.00 |
| Composite adverse neonatal outcome ∫ | 2 (3.4) | 1 (5.6) | 0.56 |
| NICU admission n(%) | 5 (8.5) | 2 (11.1) | 0.66 |
| Gestational age at birth  <32 wk n(%)  <34 wk n(%)  <37 wk n(%) | 3 (5.1)  5 (8.5)  9 (15.3) | 1 (5.6)  3 (16.7)  4 (22.2) | 0.94  0.38  0.49 |
| Birthweight  <2500g n(%)  <1500g n(%) | 9 (15.3)  2 (3.4) | 5 (27.8)  1 (5.6) | 0.23  0.56 |

* Deceased children are excluded in this number

† Maternal and neonatal characteristics in follow-up participants are compared to maternal and neonatal characteristics in the group that was loss-to follow-up due to mortality, unavailable contact data to approach parents or parents not willing to participate. The first p-value represents this comparison. The second p-value* gives the same comparison without the deceased children.

‡ parental education (high-middle-low): “low level” (total years post-elementary schooling:<6) if at least one of the parent has a low level of education (but not if one parent is highly educated), “middle level” (total years post-elementary schooling: 6-8)if both parents have middle level of education, “high level” (total years of post-elementary schooling:>8) if at least one pare
